# Exploration of the relationship between social cognition and PTSD using data from the ALSPAC cohort

**DOI:** 10.3389/fpsyt.2026.1824710

**Published:** 2026-09-14

**Authors:** Chantelle Wiseman, Sarah Sullivan, Stan Zammit

**Affiliations:** Department of Population Health Sciences, University of Bristol, Bristol, United Kingdom

**Keywords:** ALSPAC, cohort study, mentalization, theory of mind, trauma

## Abstract

**Background and hypothesis:**

Previous studies have shown an association between social cognition (emotion recognition, social perception, attributional style, and mentalization) and post-traumatic stress disorder (PTSD), but there have been no longitudinal studies on this topic. We hypothesize that childhood social cognitive differences are associated with subsequent trauma exposure and PTSD.

**Study design:**

Data from the Longitudinal Study of Parents and Children (ALSPAC) were used. The Social Communications Disorder Checklist (SCDC) at age 7.5 years, the Diagnostic Analysis of Non-Verbal Accuracy Scale (DANVA) Receptive Facial Expressions Subtest at 8 years, and the Emotional Triangles task at 13.5 years were used. Outcome measures were trauma ages 14–23 years using the LEC-5 and screening positive for PTSD at 23 years of age using the PCL-5. Logistic regression analyses were used and adjusted for sex, socioeconomic status, verbal IQ, and autism diagnosis for the trauma outcome and, additionally, childhood trauma ages 0–10 years for the PTSD outcome. A sensitivity analysis examined whether an association remained with adolescent/adult trauma after excluding those with childhood trauma.

**Study results:**

Global social cognition differences measured via the SCDC were associated with trauma in the main and sensitivity analyses (OR_adj_ 1.09, 95% CI 1.08, 1.11, *p*-value <0.001) as were errors on the Triangles task (OR_adj_ 1.04, 95% CI 1.03, 1.06, *p*-value <0.001). The SCDC was also associated with PTSD (OR_adj_ 1.15, 95% CI 1.12, 1.18, *p*-value <0.001) as were errors on the Triangles task (OR_adj_ 1.16, 95% CI 1.13, 1.20, *p*-value <0.001). No association was found with the DANVA task and the outcomes measured.

**Conclusions:**

These results support a temporal relationship between social cognitive variation and subsequent trauma and PTSD.

## 1 Introduction

Social cognition refers to the understanding of the social environment as well as processing of this information and the effect it has on behavior (1). It is a key domain of neurocognition (2). It includes a number of different capabilities (3). There are several different approaches to the categorization of social cognition: the three-factor structure seems to be the most consistent and replicable, especially in clinical populations (4, 5). This consists firstly of the lower-level abilities emotion recognition/processing and social perception. Emotion recognition is the ability to attribute emotions to static and dynamic configurations of facial movements as well as vocal prosody (6). Social perception refers to recognizing and interpreting social cues in others and relies on an understanding of the rules, roles, and goals that are the norms for that society (6). The next level of complexity is attributional style or bias, which is how causes of events are interpreted (6). Finally, the highest level, social cognitive ability, is mentalization or theory of mind. This is the ability to identify mental states in oneself and others and the use of this knowledge to explain behaviors (7).

The social cognitive theory of post-traumatic stress disorder (PTSD) hypothesizes that variations in social cognition increase the likelihood of PTSD developing after a traumatic event (8). The proposed pathway is that insecure attachment in childhood affects the development of the child’s social cognition. As a result, the child is more likely to experience and be more vulnerable to the effects of trauma. This results in an increased risk of PTSD (8). Social cognitive differences are hypothesized to lead to outcomes of trauma and PTSD through a number of mechanisms (8). Firstly, variations in social cognition could result in increased trauma exposure and, therefore, a higher risk of PTSD due to the increased amount of trauma (particularly interpersonal) experienced. In addition, the threshold for what is considered to be traumatic may also be lowered, also increasing the likelihood of PTSD occurring. Reduced social support during the peri-trauma period is another proposed pathway.

Prior reviews have shown that social cognition is altered in people with PTSD compared to controls (9–11). Specifically, affective empathy and mentalization (including affective and cognitive theory of mind) differ, but there is heterogeneity in the results (9–11). The vast majority of primary studies have been either cross-sectional or case–control designs—for example, we found an increase in mentalizing differences in people with PTSD compared to controls in one of the largest studies to date (*n* = 197) (12). We were unable to test the causal direction given the single time point for the collection of data. As far as we are aware, no studies have used longitudinal data to examine whether social cognitive differences precede the onset of trauma and PTSD.

We used data from the Avon Longitudinal Study of Parents and Children (ALSPAC) study to examine whether problems with social cognition in childhood increase the odds of subsequent trauma and of screening positive for PTSD at 23 years of age.

## 2 Materials and methods

### 2.1 Ethical approval

Ethical approval for the study was obtained from the ALSPAC Ethics and Law Committee and the Local Research Ethics Committees. The authors assert that all procedures contributing to this work comply with the ethical standards of the relevant national and institutional committees on human experimentation and with the Helsinki Declaration of 1975, as revised in 2013.

### 2.2 Study sample: The ALSPAC cohort

We used data from the ALSPAC cohort (13–15). Pregnant women resident in Avon, UK, with expected dates of delivery between April 1, 1991 and December 31–1992 were invited to take part in the study. The initial number of pregnancies enrolled was 14,541. There were 14,062 live births and 13,988 children who were alive at 1 year of age. A later recruitment round at age 7 years sought to find more of the mother–child dyads eligible from the study population, leading to a maximum sample of 15,247 women enrolled in the study (13). The total sample size for analyses using any data collected after the age of 7 years is therefore 15,447 pregnancies, resulting in 15,658 fetuses. Of these, 14,901 children were alive at 1 year of age.

Of the ALSPAC participants, 1,653 had data on all of the variables in this study (complete case). **Figure 1** shows the numbers of participants with data available on each variable. Our imputed sample was 4,086 participants.

**Figure 1:**
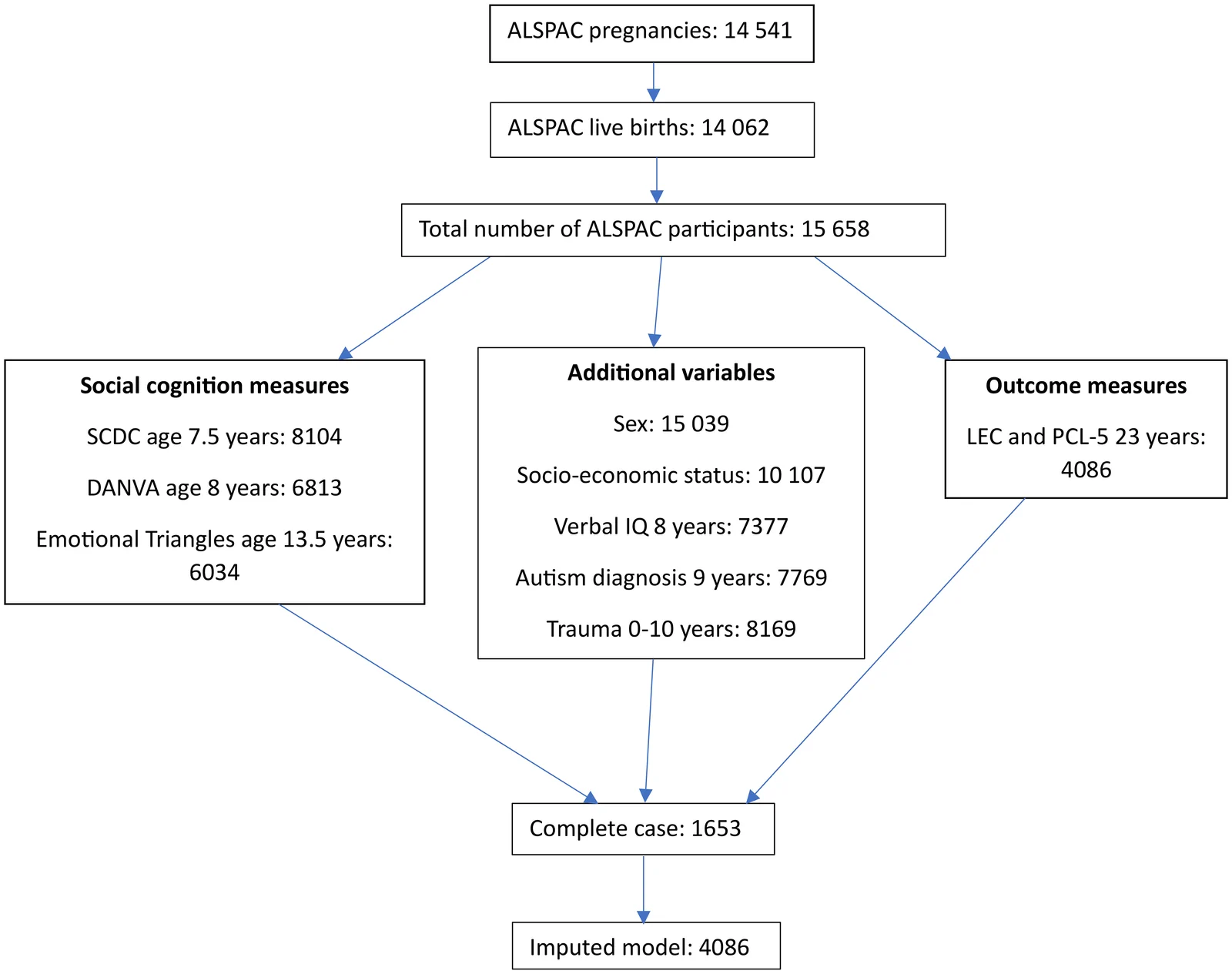
Flow chart of the study participants and number of participants for whom data are available on each variable.

Study data were collected and managed using REDCap electronic data capture tools hosted at the University of Bristol (16). REDCap (Research Electronic Data Capture) is a secure, web-based software platform designed to support data capture for research studies.

Please note that the study website contains details of all the data that are available through a fully searchable data dictionary and variable search tool and reference the following webpage: http://www.bristol.ac.uk/alspac/researchers/our-data/.

### 2.3 Variables

#### 2.3.1 Social cognition variables

Three childhood/adolescent social cognition measures were used: the Social and Communications Disorder Checklist (SCDC) (17, 18), the Diagnostic Analysis of Non-Verbal Accuracy Scale (DANVA) Receptive Facial Expressions Subtest (19), and the Emotional Triangles task (20). All variables were standardized for ease of comparison using the “egen” STATA command for the complete case sample and then imputed. A higher score in each variable is indicative of greater challenges with social cognition.

##### 2.3.1.1 Social and Communications Disorder Checklist

The Social and Communications Disorder Checklist (SCDC), originally called the Skuse questionnaire (17), was first designed to measure social cognitive skills in people with Turner syndrome (17). This questionnaire was completed by the parents reporting on the child’s social cognition at 91 months (7.5 years). The SCDC questionnaire measures 12 aspects of social behavior and communications, namely (17): awareness of the feelings of others, recognizing overt emotions in others, recognizing the effects of own behavior on close individuals, disruptive behavior, demanding behavior, inability to be reasoned with when upset, poor social skills, reading body language, behavior when out and about, offensive behavior, obedience, and ability to follow commands (17, 18). Parents report whether the statements are not true, sometimes true, or often true of their study child. This measure has high internal consistency and test–retest reliability (18). We consider it to be a global measure of social cognition.

##### 2.3.1.2 DANVA task: receptive facial expressions subtest

The Diagnostic Analysis of Non-verbal Accuracy Scale (DANVA) is a measure of emotion recognition, consisting of seven subtests assessing receptive and expressive non-verbal information (19). In the ALSPAC cohort, the participants completed the Receptive Facial Expressions Subtest of the DANVA at 8 years of age. The task consists of 24 trials. Each trial shows a posed photo of a child’s face and the participant has to select whether they are happy, angry, fearful, or sad. We used a continuous variable that described the number of incorrect responses across all trials, irrespective of emotion. The DANVA has shown good internal consistency (coefficient alpha of 0.88) and a test–retest score of 0.84 in a community sample (18). This is a measure of lower-order social cognitive ability: emotion recognition.

##### 2.3.1.3 Emotional triangles task

This emotion recognition and mentalizing task (20, 21) was completed by the ALSPAC participants at 13.5 years of age. The task consists of participants watching short video clips of an animated triangle interacting with a circle (20). The participants were asked questions about the degree a particular emotion is shown by the animated triangle and how animated or living it is, both on a scale from 0 to 5. We produced a score of errors with this task by inverse coding the trials where the participants were asked about the correct emotion and tallying the scores. We scored the task in this way, which differed from the original task scoring, to produce a measure of inaccuracy.

### 2.4 Outcome variables

#### 2.4.1 Trauma

##### 2.4.1.1 Modified Life Events Checklist for DSM-5

The Life Events Checklist for DSM-5 (LEC-5) (22) is a questionnaire measure of lifetime exposure to traumatic experiences. A simplified version was administered to ALSPAC participants that included only six items covering whether the participant had experienced a serious accident or fire, had experienced a serious attack or threat of attack, had experienced sexual assault, witnessed a sudden violent death, experienced the sudden, unexpected death of someone close to them, or any other extremely stressful event. The trauma was then coded by the ALSPAC team. If a trauma was endorsed, the participants were then specifically asked if it involved threatened death, death of another, sexual assault, or severe injury and separately whether it involved learning of the death of a loved one. The age of the participant when the event occurred was also recorded.

We derived a binary variable for this measure for those who endorsed any of these exposures. A total of 4,086 participants completed this questionnaire. We excluded from the analyses participants exposed to any trauma measured on the LEC-5 prior to 14 years to minimize reverse causation (as the social cognition measures were taken between 7 and 13.5 years of age).

#### 2.4.2 PTSD

The PTSD Checklist for DSM-5 (PCL-5) questionnaire was completed by participants who endorsed any trauma exposure on the LEC-5 at age 23 years. The PCL-5 consists of 20 items which measure past-month symptoms of PTSD based on the DSM-5 diagnostic criteria. Items are scored from 0 to 4 in increasing severity, and a total score was derived by summing these (range 0–80).

We coded individuals as screening positive for PTSD if they reported a trauma exposure measured via the LEC-5 and they had a score of 33 or greater on the PCL-5 (23).

### 2.5 Covariates

Variables which are associated with social cognition and PTSD were measured and adjusted for as confounders.

#### 2.5.1 Sex

There are some sex-based differences in social cognition (24, 25). Women are more likely than men to develop PTSD (26). Sex is recorded at birth in the ALSPAC cohort.

#### 2.5.2 Intelligence quotient

Verbal IQ is associated with both social cognitive ability (27, 28) and PTSD (29). We used data from The Wechsler Intelligence Scale for Children verbal IQ score at 8 years of age.

#### 2.5.3 Socioeconomic status

Social cognition is associated with education and socio-economic status (SES) (27). SES is also associated with the likelihood of developing PTSD (30). We used mother’s social class measured during pregnancy as a marker of SES.

#### 2.5.4 Autism

One of the defining features of autism is differences in social behavior, particularly mentalization (31). Autism is also associated with a higher risk of PTSD (32). We used a binary measure in ALSPAC of maternal report of an autism spectrum diagnosis for the child, measured at 9 years of age.

#### 2.5.5 Childhood trauma

Trauma is necessary for the development of PTSD, and childhood traumatic and adverse experiences are associated with an increased likelihood of problems with social cognition (33, 34).

A measure of trauma exposure between 0 and 10 years of age was derived from multiple questions from assessments taken contemporaneously covering physical, sexual, and emotional abuse, bullying, emotional neglect, and exposure to domestic violence. The participants were coded as experiencing childhood trauma if they or their caregiver endorsed any of the questions relating to these traumas between ages 0 and 10 years of age. The participants were coded as non-exposed if they or their caregiver had not endorsed any of the questions and they had participated in at least 50% of assessments. For further details on how this measure was derived, see Croft et al. (2018) (35).

We used this variable both as a confounder (when examining the relationship between social cognition and PTSD) and as an exclusion criteria (for the sensitivity analysis examining the relationship between social cognition and trauma) to minimize reverse causation.

### 2.6 Statistical methods

Stata version 18 was used for the analyses (36).

### 2.7 Analytical techniques

#### 2.7.1 Correlation

Spearman’s correlation was used to examine the correlation between the social cognition measures.

#### 2.7.2 Regression analyses

Logistic regression was used to determine whether childhood social cognitive ability was associated with trauma and PTSD. Unadjusted and adjusted odds ratios, 95% confidence intervals, and *p*-values are presented to describe the odds of outcome per standard deviation increase in the exposure variable. Confounders for trauma exposure as an outcome were sex, verbal IQ, SES, and autism. Additionally, we also adjusted for childhood trauma (ages 0–10 years) when examining PTSD as the outcome.

#### 2.7.3 Sensitivity analysis

We undertook a sensitivity analysis to minimize reverse causation, i.e., to minimize the possibility that early-life trauma exposure affected participant social cognition scores in the social cognition assessments measured between 7.5 and 13.5 years of age. To do this, we restricted the sample for analyses of the association between social cognition and trauma ages 14–23 years to participants who did not have a trauma recorded between the ages of 0 and 10 years of age.

#### 2.7.4 Missing data

Multiple imputation using chained equations was completed using the “ice” command in STATA. We ran multiple imputation in a dataset that had complete case information on the outcome variables of trauma and PTSD, which were both measured at age 23 years (*n* = 4,086). A total of 20 cycles of imputation were carried out and combined using Rubin’s rules. Imputation was of the social cognition variables and covariates. All social cognition, outcome, and confounder variables were included in the imputation model. Additional auxiliary variables were used to build the imputed data set based on variables associated with missingness and the variables of interest. These included variables on separation anxiety, bonding, additional autism measures, personality, locus of control, temperament, neuroticism, socio-economic status, cruelty in the home, and maternal depression and substance use during pregnancy.

## 3 Results

### 3.1 Demographics

A total of 1,653 participants formed the complete case sample who had data on all variables. There were 4,086 participants in the imputed sample. The participants in the complete case sample were different from the rest of the ALSPAC cohort. The complete case sample had fewer men, were of a higher socioeconomic status, and were less likely to have experienced child abuse (see **Table 1**). The remainder of the results presented are from the imputed data.

**Table 1:** Differences in socio-demographic characteristics and childhood trauma between the complete case sample, the rest of the ALSPAC cohort, and the imputed sample.

Characteristic	Missing from complete case sample, *N* (%)	Included in complete case sample, *N* (%)	*P*-value	Included in imputed sample, %
Male sex	7,062 (52.8%)	629 (38.1%)	<0.001	34.4
Non-manual maternal social class	6,648 (78.6%)	1450 (87.7%)	<0.001	84.7
Maternal smoking in pregnancy	3,131 (26.9%)	175 (10.6%)	<0.001	16.0
Trauma ages 0–5 years	2,362 (27.0%)	327 (19.8%)	<0.001	21.9

### 3.2 Correlation between the social cognition measures

There was weak correlation between social cognition measured via the SCDC, DANVA, and Triangles measures, with Spearman’s rho ranging between 0.03 and 0.12 (see **Table 2**).

**Table 2:** Correlation between the social cognition measures.

Measure	SCDC	DANVA errors
SCDC	1.00	–
DANVA errors	0.09 *p* < 0.001	1.00
Emotional Triangles errors	0.03 *p* < 0.001	0.12 *p* < 0.001

### 3.3 Social cognition and trauma

**Table 3** shows that variation in social cognition measured using the SCDC was associated with increased odds of subsequent trauma exposure, and this relationship was unchanged after adjusting for confounders (OR_adj_ 1.09, 95% CI 1.08, 1.11, *p*-value <0.001). There was also an increase in odds of trauma exposure with errors on the Triangles task that persisted after adjusting for confounders (OR_adj_ 1.04, 95% CI 1.03, 1.06, *p*-value <0.001). These findings reach statistical significance but are a modest increase per standard deviation.

**Table 3:** Odds ratios, 95% confidence intervals and p-values describing the relationship between social cognition and trauma.

	Crude			Adjusted^a^		
Measure	OR	95% CI	*P*-value	OR	95% CI	*P*-value
SCDC	1.10	1.08, 1.11	<0.001	1.09	1.08, 1.11	<0.001
DANVA errors	1.00	0.99, 1.01	0.992	1.00	0.98, 1.01	0.764
Emotional Triangles errors	1.05	1.04, 1.07	<0.001	1.04	1.03, 1.06	<0.001

^a^ Adjusted for confounders: sex, verbal IQ, SES, and autism.

Crude and adjusted results shown.

### 3.4 Social cognition and PTSD

We found increased odds of screening positive for PTSD in those with higher scores on the SCDC at 7.5 years of age (OR_adj_ 1.15, 95% CI 1.12, 1.18, *p*-value <0.001) and a greater number of errors on the Triangles task at 13.5 years of age (OR_adj_ 1.16, 95% CI 1.13, 1.20, *p*-value <0.001) (see **Table 4**). These findings again reach statistical significance but are a modest increase per standard deviation.

**Table 4:** Odds ratios, 95% confidence intervals and p-values describing the relationship between social cognition and screening positive for PTSD.

	Crude			Adjusted^a^		
Measure	OR	95% CI	*P*-value	OR	95% CI	*P*-value
SCDC	1.18	1.15, 1.21	<0.001	1.15	1.12, 1.18	<0.001
DANVA errors	1.03	1.00, 1.06	0.027	1.02	0.99, 1.05	0.195
Emotional Triangles errors	1.22	1.18, 1.25	<0.001	1.16	1.13, 1.20	<0.001

^a^ Adjusted for confounders: sex, verbal IQ, SES, autism, and childhood trauma ages 0–10 years.

Crude and adjusted results shown.

### 3.5 Sensitivity analyses restricting the sample to those without childhood trauma

We repeated the regression analyses examining whether social cognition was associated with adolescent/adult trauma exposure in a sensitivity analysis where we restricted the sample to those who did not experience a trauma by age 10 years. Association with the SCDC remained similar (OR_adj_ 1.08, 95% CI 1.06, 1.11, *p*-value <0.001). The odds ratio for errors on the Triangles task continued to show an association, but this was slightly reduced (OR_adj_ 1.02, 95% CI 1.00, 1.04, *p*-value 0.025) (see **Table 5**).

**Table 5:** Odds ratios, 95% confidence intervals, and *p*-values describing the relationship between social cognition and trauma restricted to those without childhood trauma.

	Crude			Adjusted^a^		
Measure	OR	95% CI	*P*-value	OR	95% CI	*P*-value
SCDC	1.09	1.06, 1.11	<0.001	1.08	1.06, 1.11	<0.001
DANVA errors	1.00	0.98, 1.02	0.686	1.00	0.98, 1.02	0.769
Emotional Triangles errors	1.03	1.01, 1.06	0.001	1.02	1.00, 1.04	0.025

^a^ Adjusted for confounders: sex, verbal IQ, SES, autism.

Crude and adjusted results are shown.

## 4 Discussion

### 4.1 Summary of findings

In this study, using data from a large population cohort, we found an increase in the odds of adolescent/early adulthood trauma exposure with increasing social cognitive challenges in childhood measured using the SCDC questionnaire and errors on the Triangles task. We also found an association between childhood social cognition using the same measures and screening positive for PTSD in early adulthood. After adjusting for confounders, we did not find an association between the emotion recognition task, the DANVA, and outcomes of trauma or PTSD.

### 4.2 Strengths and limitations

To our knowledge, this is the first longitudinal study examining whether social cognitive differences are associated with subsequent exposure to trauma and screening positive for PTSD. It used contemporaneously collected data from a large population study. This has allowed us to demonstrate some confidence in the temporality of the relationship, more so than from previous studies completed at a single time point. Validated measures were used for the social cognition and PTSD variables (and the trauma variable was a modified version of a validated tool). Imputation models were used to minimize bias from attrition and to increase the power and precision of the findings. A sensitivity analysis was used for the outcome of trauma, restricting the sample to those who had not experienced childhood trauma to reduce the likelihood of reverse causation explaining the results.

However, the study must be viewed in light of several limitations. Firstly, in terms of the measures used—a PTSD screening tool, the PCL-5, was used to identify participants with PTSD, rather than a clinical assessment. While adequate validity of the PCL-5 has been demonstrated in many settings, the cutoff score maximizing agreement with gold-standard clinical assessments such as the CAPS-5 varies across studies (37). It is possible, therefore, that some of our sample cases may not have met the clinical diagnostic criteria, and indeed we observed a higher proportion of PTSD than the 7% lifetime prevalence reported in previous studies (38). The LEC-5 version available to us was modified and did not include the full range of traumas from the original, validated questionnaire. This could have affected the accuracy of the trauma measures. In addition, the PCL-5 was only completed if a participant endorsed a trauma on the LEC-5, but if they had experienced more than one trauma, it would not be possible to identify which traumatic event precipitated the PTSD experiences.

Measurement error in any of our social cognition or outcome measures would have led to an underestimate of associations if non-differential in nature but could have overestimated effect sizes if differential. Error in our confounder measures would have led to underestimation of confounding, and this might be particularly true for autism as we used a binary parent-report measure of autism diagnosis rather than a quantitative measure of autistic traits. As variations in social communication and interactions are core features of autism spectrum disorders, our approach will have minimized problems of over-adjustment.

We used a different technique from the published method for scoring the Triangles errors task. We produced a score of errors with the task so that we could compare with the other measures of social cognition, i.e., a higher score in all measures was indicative of challenges with social cognitive skills. Previous researchers have used scoring techniques which differ from the published methodology for the Emotional Triangles Task (21). We recognize that this is not the published method of scoring this task and could affect the accuracy of the results.

In addition, the participants in the ALSPAC cohort are from one geographical area and are wealthier and more ethnically homogenous than the UK as a whole (14), which could affect the generalizability of findings.

The imputed sample was approximately 4000, but the ALSPAC cohort consists of over 15,000 participants; it is possible therefore that attrition bias could affect the findings. We decided to impute only those participants who had completed the outcome measures at age 23 to increase the reliability of the imputation model as there was more data available on other measures for the imputation model.

We found little correlation between our social cognition measures. This could be due to the instruments measuring different aspects of social cognition.

We attempted to minimize any overlap between the timings of the main study variables (the social cognition, trauma, and PTSD measures) to demonstrate temporality and strengthen our study findings. The social cognition measures were taken at 7–13.5 years of age, the LEC-5 period covered traumas prior to 23 years of age (and we excluded participants where the trauma occurred below 14 years of age), and the PCL-5 measured symptoms of PTSD at 23 years of age. We also used a variable of childhood trauma ages 0–10 years as a confounder for the regression model with the outcome of a positive screen for PTSD and to exclude participants from a sensitivity analysis restricting the regression model with trauma to those without trauma before the age of 10 years. Unfortunately, there may still have been some individuals affected by trauma that occurred prior to the social cognition measures included in the sensitivity analysis, as the social cognition measures were completed between 7 and 13.5 years of age and the secondary trauma variable only excluded trauma between 0 and 10 years of age.

### 4.3 Comparison with previous literature

It has been hypothesized that variations in social cognition increase the risk of PTSD (8). The social cognitive theory of PTSD hypothesizes that insecure attachment in childhood affects the development of the child’s social cognition; the child is then more likely to experience and be more vulnerable to the effects of trauma, resulting in an increased risk of PTSD (8). We found an association between the SCDC and errors on the Triangles task, but not the DANVA emotion recognition task and outcomes of trauma and screening positive for PTSD. This builds on previous work using a case–control study design where we found that a self-report measure of mentalization differences (a higher-order social cognitive ability), but not other social cognition measures, was associated with PTSD (12).

Previous cross-sectional and case–control studies have found associations between social cognition and PTSD, although the findings are heterogenous and such study designs have not been able to tease out the direction of effects (9–11). A large effect size (SMD -0.81) has been found between PTSD groups and mentalizing differences compared to controls in one systematic review and meta-analysis (11). The social cognition measures that we found to be associated with PTSD in this study (SCDC score and errors on the Triangles task) provided a broad coverage of social cognitive abilities. We did not find an association between the DANVA (a lower-order emotion recognition task) and PTSD. As far as we are aware, there are no other longitudinal studies examining the temporal sequence between social cognition, trauma, and PTSD. Other studies have looked at the association between social cognition measures and other psychiatric outcomes. Associations have been found between social cognition in childhood and subsequent depression, internalizing and externalizing problems and eating disorders in adolescence, as well as mediating between emotional dysregulation and disordered eating and self-harm (39–42). It is plausible that social cognitive changes may be associated with psychiatric conditions more broadly. Other studies have failed to find an increase in likelihood with scores on the DANVA at 8 years and outcomes of psychotic-like experiences and substance misuse (43, 44).

### 4.4 Interpretation of findings

This study has found an association between parent-reported social cognitive differences and scores on the Emotional Triangles task during childhood and outcomes of adolescent trauma and screening positive for PTSD at 23 years of age. This is the first study (that we are aware of) which looks at the temporal relationship between differences in social cognition, trauma, and PTSD. The size of the findings is modest compared to previous work in case–control and cross-sectional study designs, but such studies may have overestimated effect sizes as a result of reverse causation (i.e., PTSD affecting social cognition). Potentially, a bidirectional relationship between social cognition and PTSD exists (6, 45). Although we found a temporal relationship between measures showing differences with social cognition and outcomes of trauma and PTSD, the modest size of the odds ratios and potential measurement issues could limit the implications of our study findings.

Differences in mentalizing are hypothesized by Sharp et al. (2012) to increase the likelihood of PTSD by several mechanisms (5). These include increased likelihood of interpersonal trauma, reduced threshold for what is considered traumatic, and reduced social support in the peri-trauma period. We have also hypothesized that hypermentalizing and distraction resulting in avoiding processing one’s own trauma could be another mechanism for this relationship (45).

Trauma does not just cause PTSD but has causal effects on the development of other psychiatric conditions as well (46). Further research is needed to establish whether an increase in trauma as a consequence of differing social cognition could increase a broad range of psychiatric conditions.

## 5 Conclusion

This longitudinal study has found an association between social cognition differences in two out of three measures taken in childhood and adolescence and screening positive for PTSD at 23 years of age. These findings build on previous studies which have found mentalization differences in PTSD groups compared to controls. We have established a temporal relationship between variation in social cognition (using two broad measures) and outcomes of trauma and a PTSD positive screen.

## Data Availability

Publicly available datasets were analyzed in this study. This data can be found here: http://www.bristol.ac.uk/alspac/researchers/our-data/. Please note that the study website contains details of all the data that is available through a fully searchable data dictionary and variable search tool.
